# Cytoreductive Surgery and Hyperthermic Intraperitoneal Chemotherapy (CRS + HIPEC): an initial experience at a Tertiary care Hospital in Pakistan

**DOI:** 10.12669/pjms.41.12.11686

**Published:** 2025-12

**Authors:** Sibgha Aimon, Hadi Mohammad Khan, Ushna Khan, Muslim Atiq

**Affiliations:** 1 Sibgha Aimon, Department of General Surgery, Shifa International Hospital, Islamabad, Pakistan; 2 Hadi Mohammad Khan, Department of General Surgery, Shifa International Hospital, Islamabad, Pakistan; 3 Ushna Khan, Department of General Surgery, Shifa International Hospital, Islamabad, Pakistan; 4 Muslim Atiq, Department of Gastroenterology, Shifa International Hospital, Islamabad, Pakistan

**Keywords:** Cytoreductive Surgery (CRS), Hyperthermic Intraperitoneal Chemotherapy (HIPEC), Low Grade Appendiceal Mucinous Neoplasm (LAMN)

## Abstract

**Objective:**

Cytoreductive surgery (CRS) and Hyperthermic Intraperitoneal Chemotherapy (HIPEC) is evolving technique for advance stage malignancies. Studies have shown that CRS and HIPEC improve survival, however initial published results were not promising in view of high morbidity 20% and mortality 4.8%, hence it has yet to be standard of care for certain malignancies and further trials are awaited. This study presents outcomes of HIPEC in a Single center of a developing country.

**Methodology:**

This retrospective cross-sectional study was conducted in Shifa International Hospital, Islamabad, Pakistan. Data is collected from January 2019 to December 2023. Patients who underwent CRS and HIPEC for curative intent were included in the study. Quantitative analysis is represented by mean and standard deviation, qualitative analysis is represented by frequency and percentage.

**Results:**

Fifty patients were included. Out of these 17 (34%) were Male, and 33(66%) Female. Ovarian carcinoma was most common pathology 36%, Adenocarcinoma Colon and Low grade Appendiceal Mucinous Neoplasm 24%. No 30-day mortality was observed in our study. Recurrence was observed in 16 (32%) patients. Patient with poor tumor biology had a higher rate of recurrence (p= 0.003). Mean Overall Survival among patient who underwent HIPEC was 45±3 months. For patients who had LAMN, mean survival was 50±5 months which was significantly better than rest of pathologies.

**Conclusion:**

Cytoreduction and HIPEC offers a hope for patients with advance malignancy, however patient selection for undergoing HIPEC is of utmost importance.

## INTRODUCTION

Cytoreductive surgery (CRS) and Hyperthermic Intraperitoneal Chemotherapy (HIPEC) emerged in 1990s as a treatment option for patient with peritoneal carcinomatosis especially from ovarian or gastrointestinal origin malignancies, without extra peritoneal disease. Peritoneal carcinamatosis has poor prognosis with a mean survival of approximately six months only. Earlier studies on Cytoreduction and HIPEC reported high morbidity and mortality, but with further trials, advancement in surgical technique and perioperative care, significant improved outcomes reported.^1^ A study performed by Foster et al reported only 1.1% 30 days mortality which is lower in comparison to the other surgical procedures routinely being performed without the intention of HIPEC and cytoreduction including hepatectomy and Whipple’s procedure.^2^ With respect to survival benefit, multiple studies have been conducted but no consensus is made.

In 2021, PRODIGE-7 trial showed no survival benefit of HIPEC in patients with colorectal peritoneal metastasis and recommended cytoreductive surgery only as intention for cure,^3^ leading to further trials for evaluation of outcomes and safety of HIPEC. However, Ji Li et at analyzed the data of colorectal cancer patients who underwent cytoreduction and HIPEC and published a systematic review and meta-analysis supporting HIPEC with survival benefit in colorectal patients with peritoneal metastasis.^4^ Similarly, Tinsley et al in recent ASCO meeting presented their findings on outcomes of HIPEC in colorectal patients who have peritoneal metastasis, suggesting better overall survival use of Oxaliplatin as a chemotherapeutic agent in HIPEC.^5^ For advanced ovarian cancer, with systemic chemotherapy, HIPEC and CRS has showed to improved progression free and overall survival.^6^ With regards to gastric malignancies, CRS and HIPEC has showed to improve survival however with high morbidity 20% and mortality 4.8% rate it has yet to be standard of care and further studies are awaited.^7^

## METHODOLOGY

This was a single center Retrospective observational study, done at Shifa International Hospital Islamabad.

### Inclusion criteria:

We included patients who underwent Cytoreductive Surgery and HIPEC for Primary and Secondary peritoneal carcinomatosis between January 2019 and December 2023.

### Exclusion criteria:

Patient who only underwent cytoreductive surgery and not proceeded for HIPEC, and had Completeness of Cytoreduction (CC) score of two or more were excluded from study.

### Ethical Approval:

Institutional Review Board (IRB) approval (IRB # 373-23) was obtained on July 8, 2024 and data collection started.

All cases were discussed in multidisciplinary team meeting which included a surgeon, an oncologist, and a pathologist, and after a final consensus decision, patient proceeded for the procedure. All the data was documented in patient confidential records. The data was recorded in a questionnaire and analyzed using SPSS version 26.

All patients underwent Hyperthermic Intraperitoneal chemotherapy with the “Closed abdomen technique”, using two Inflow and two outflow drains. Only one patient underwent laparoscopic cytoreduction because of very low PCI. The machine used for this procedure was procured through Therma Solutions. The chemotherapy administered is dispensed from pharmacy after order from oncologist and the dose is calculated according to Body Surface Area of patients and then administered, after Cytoreduction is performed, at 42ºC for 90 minutes. After completion of HIPEC abdomen is again opened, thoroughly washed and bowel continuity restored (if any resection of bowel was performed during cytoreduction) followed by drains placement and closure of fascia and subcutaneous tissue and skin. Details on quantitative variables like age, body mass index, body surface area, peritoneal carcinomatosis index, completion cytoreduction score, chemotherapy used in HIPEC, duration of surgery, and duration of ICU stay, Peritoneal Carcinomatosis Index (PCI) and CC score as well as histopathology were collected. Quantitative variables including age, BMI and BSA, duration of surgery, hospital stay, ICU stay is represented as mean and standard deviation. Qualitative variables are presented in frequency and percentage. Statistical significance between variables and recurrence is calculated using Chi square and is represented as p-value. Survival analysis represented using Kaplan Meier curve. For analysis, adenocarcinoma appendix was included with Colonic Carcinoma, and Low Grade Appendiceal Mucinous Neoplasm was analyzed separately.

## RESULTS

**Fig.1:**
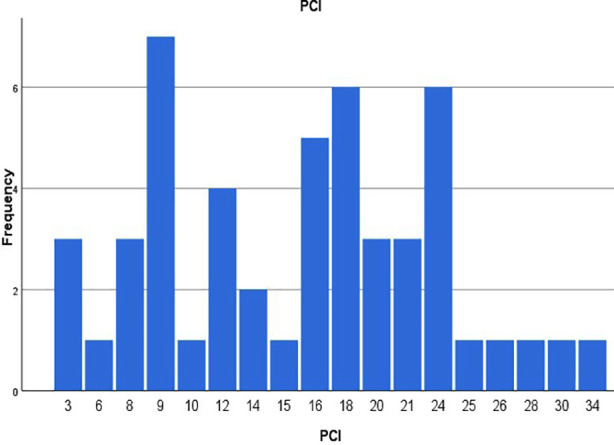
A Histogram representing frequency of Peritoneal Carcinomatosis Index (PCI).

A total of 54 patients underwent CRS and HIPEC., out of which four were excluded due to palliative intent of procedure. On 50 remaining patients’ final analysis was performed. Of these, 17 (34%) were Male, and 33(66%) Female. With regards to comorbid conditions, 10 (20%) were diabetic, 11 (22%) Hypertensive, ASA was three in 42 (84%), ECOG status was 0 in 44 (88%) patients. Mean Peritoneal Carcinomatosis Index (PCI) was 16.02 ± 7.32 (Fig.1). CC-0 and CC-1 was achieved in 41 and nine patients respectively. Quantitative variable data is represented in Table-I.

**Table-I:** Quantitative variables. Body Mass Index (BMI), Body Surface Area (BSA), Peritoneal Carcinomatosis Index (PCI).

Variable	Mean ± SD
Age (Years)	49.37±11.2
BMI (Kg/m2)	27.19±5.5
BSA (m2)	1.79±0.20
PCI	16.02±7.32
Duration of Surgery (Minutes)	440±85.8
ICU stay (Hours)	32.08±22.9
Hospital stays (Days)	7.2±1.4

Initial diagnosis was Ovarian carcinoma in 18 patients (36%), for which CRS and HIPEC was performed, followed by Adenocarcinoma Colon 12(24%), Low grade Appendiceal Mucinous Neoplasm - LAMN 12 (24%), and Pseudomyxoma Peritonei 6 (12%). Neoadjuvant Chemotherapy was administered in 28 (56%) and one patient received neoadjuvant radiation therapy. Tumor markers including CA 125, CEA and CA 19-9 were elevated in 31(62%) patients. Chemotherapy used during HIPEC was Mitomycin in 31 (62%) patients, and Cisplatin in 18 (36%) patients.

In 30 days, no complication was observed in 44 (88%) patients. Total two of these patients had Clavien Dindo class 3b complications, and one patient developed class IV complication. Only one patient was reoperated for bleeding which was controlled successfully, one patient had Enterocutaneous Fistula, one patient develops Non-ST Elevation Myocardial Infarction (NSTEMI) postoperative which was managed conservatively taking cardiology team on board.

No patient died within the 30 days post op. Cumulative disease recurrence rate was 32%. Patient with poor tumor biology at the time of initial presentation, had a higher rate of recurrence (p= 0.003). LAMN had a recurrence rate of (16%) as compared to adenocarcinoma colon (75%), and ovarian cancer (22%). There was no significance of PCI to recurrence (p= 0.3), however CC score showed statistically significant value (p=0.014) indicating complete cytoreduction does play a significant role in disease recurrence. Tumor markers showed no statistical significance for recurrence.

**Fig.2:**
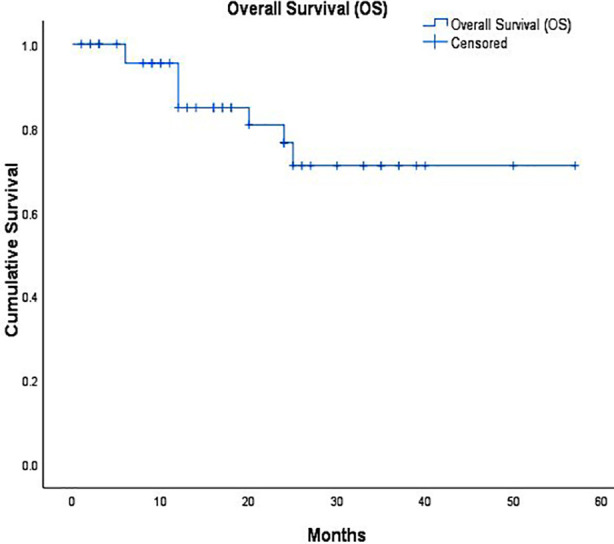
Overall Survival (OS).

**Fig.3:**
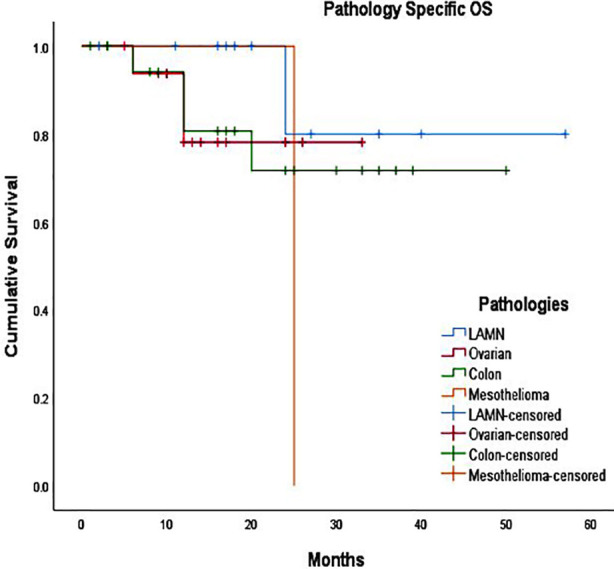
Disease specific survival. LAMN- Low Grade Appendiceal Mucinous Neoplasm. OS: Overall Survival.

Overall, survival among patient who underwent HIPEC was 45±3 months (80%). For patients who had LAMN, mean survival was 50±5 months (90%) which was significantly better than rest of pathologies. (Fig.2,3). Of these, longest surviving patient, 60 months (five years) disease free and overall survival, is of Low Grade Appendiceal Mucinous Neoplasm. Survival for Ovarian cancer 82% with mean 28±2 months, and Colonic cancer was 80%, with mean 39±4 months.

## DISCUSSION

This study presents the outcomes of 50 patients undergoing Cytoreductive Surgery (CRS) with Hyperthermic Intraperitoneal Chemotherapy (HIPEC) at a single center in a developing country, offering a valuable contribution to global literature from a lower-resource setting. Our results are acceptable with better short-term outcomes in terms of morbidity, recurrence, and overall survival, and are comparable with previous published literature.

In our study, the most common indication for CRS-HIPEC at the time of initial presentation was ovarian carcinoma (36%), followed by colorectal adenocarcinoma (24%), low-grade appendiceal mucinous neoplasm (LAMN) (24%), and pseudomyxoma peritonei (12%). These distributions correspond with literature, where ovarian and colorectal cancers remain the most frequently treated pathologies with CRS and HIPEC.^8^,^9^

The mean Peritoneal Carcinomatosis Index (PCI) in our study was 16.02 ± 7.32. In our study, PCI did not significantly correlate with recurrence (p = 0.3), however some studies emphasize PCI as a prognostic marker of recurrence,^10^,^11^ Birgisson et al used PCI score as a prognostic factor in patients with peritoneal metastasis from colorectal carcinomas being treated with CRS and HIPEC, but concluded to take other factors into account for cytoreduction^12^, as mentioned in other studies that tumor biology and response to systemic therapy may outweigh disease burden, especially in appendiceal and ovarian cancers.^13^

Cytoreduction is an extensive surgery; it not only includes local resection but multivisceral resection as well to achieve the desired CC score without significant morbidity. Complete cytoreduction (CC-0 or CC-1) was achieved in all patients in our cohort, with 82% attaining CC-0. We observed CC score significantly correlated with recurrence (p = 0.014), supporting prior findings that incomplete cytoreduction is an independent predictor of poor prognosis and recurrence.^13^

PRODIGE-7 trial outcomes suggested no significant benefit in HIPEC and used Oxaliplatin as chemotherapeutic agent for intraperitoneal chemotherapy^3^ studies conducted after PROGIDE-7 trial suggested otherwise. In our study we use Platinum based chemotherapy for patients with ovarian cancer, which over the past 10 years has been proven to be an effective agent.^14^ Studies have shown effectiveness of Mitomycin C for colorectal cancer^15^ and our results suggest promising outcomes which support recent literature.

Recurrence was observed in 32% of patients, out of these 75% recurrence was in colonic tumors, these results are comparable to study performed in United Kingdom by Hassan S et al reporting 77% recurrence in patients undergoing cytoreduction and HIPEC for advance colorectal malignancies.^16^

The overall survival in current series was 80% with mean 45 ± 3 months. Patients with LAMN had the highest survival rate of 90% with mean 50 ± 5 months, these findings are consistent with study published by Han Z et al, in which comparison was made of patients with appendiceal neoplasm undergoing HIPEC with patient undergoing surgery only and no HIPEC, long term analysis showed better survival in HIPEC group of 82.7% at five years and 76.9% at 10 years in comparison to surgery only group with five years survival of 51.3% and 10 year survival of 46.2%.^17^ Our five years analysis for ovarian cancer patients showed survival of 82%, and mortality in only 20% patients, OVHIPEC-1 trial conducted in Netherlands compared outcomes of ovarian cancer with HIPEC and showed significant survival advantage at 10 years with mortality of 88% in surgery only group and 82% in surgery + HIPEC group,^18^ this discrepancy is explained by short term follow-up in our study.

In this study, our 30-day morbidity rate of 12% and no 30-day mortality are particularly noteworthy. Sugarbaker in his articles reported morbidity of HIPEC up-to 35% and mortality up to 5%, though recent studies reported outcomes with significantly reduced morbidity (12.5 %) and mortality (2.5%),^19^ suggesting our outcomes are well within acceptable safety thresholds, despite being in a resource-limited setting. Possible reason for improved outcomes over the years is experience of surgeons with cytoreductive techniques.

A study done by Foster et al has proven the safety concern for Cytoreduction and HIPEC to be less morbid and safe procedure in comparison with other advanced surgical cases including Esophagectomy, Hepatectomy and Pancreaticoduodenectomy. He reported higher risk of infections 1.4 and 1.6 times in Esophagectomy and Pancreaticoduodenectomy respectively compared to CRS and HIPEC. Reported mortality rate in his study was of 1.1% in CRS + HIPEC, 3% in Esophagectomy, 3.9% in Hepatectomy and 2.5% in Pancreaticoduodenectomy.^2^

At the end, question remains for patients with recurrence, what happens next? Whether to attempt a repeat HIPEC surgery or manage as palliation especially in patients with ovarian cancer and LAMN which show better survival outcomes in comparison to other pathologies, is still an on-going debate requiring additional trials.

A lot of questions still remain in our minds after this initial effort of introducing and then studying HIPEC in our center. Once a desirable awareness reaches patients only then will they be able to seek this treatment option. Studying the response of different carcinomas to HIPEC±CRS remains one of the desired goals. Increased number of patients will help us to broaden the aspects of study further to include long term survival benefits as well.

### Limitations:

This data shows the initial outcomes of HIPEC, and so low number of individual pathologies for analysis. Further studies will be conducted once optimal number is achieved for long term survival analysis.

## CONCLUSION

Introducing an entirely new concept of treatment for cancer in a developing country has its own challenges. This study concludes that Cytoreduction and HIPEC is a safe treatment modality for patients with peritoneal carcinomatosis due to ovarian, appendiceal and colonic malignancies in a developing country. However, patient selection is a crucial step, and it requires a multidisciplinary team decision and effective perioperative care. Due to low number of individual pathologies, further studies will be required to establish safety profile and long-term survival benefit.

### Author’s Contribution:

**HK:** Designed the study, and did review and final approval of the manuscript.

**SA:** Did statistical analysis, drafting and editing of manuscript and responsible for the integrity of the study.

**UK:** Did data acquisition and drafting. Critical Review.

MA: Literature search, Did review and final approval of the manuscript.
